# How Can Social Media Lead to Co-Production (Co-Delivery) of New Services for the Elderly Population? A Qualitative Study

**DOI:** 10.2196/humanfactors.7856

**Published:** 2018-02-12

**Authors:** Hadi Daneshvar, Stuart Anderson, Robin Williams, Hajar Mozaffar

**Affiliations:** ^1^ School of Informatics University of Edinburgh Edinburgh United Kingdom; ^2^ Institute for the Study of Science, Technology and Innovation University of Edinburgh Edinburgh United Kingdom; ^3^ Business School University of Edinburgh Edinburgh United Kingdom

**Keywords:** social media, eHealth, mHealth, social networking, Web 2.0, health informatics

## Abstract

**Background:**

The future of health care services in the European Union faces the triple challenges of aging, fiscal restriction, and inclusion. Co-production offers ways to manage informal care resources to help them cater for the growing needs of elderly people. Social media (SM) is seen as a critical enabler for co-production.

**Objective:**

The objective of this study was to investigate how SM—private Facebook groups, forums, Twitter, and blogging—acts as an enabler of co-production in health and care by facilitating its four underlying principles: equality, diversity, accessibility, and reciprocity.

**Methods:**

We used normalization process theory as our theoretical framework to design this study. We conducted a qualitative study and collected data through 20 semistructured interviews and observation of the activities of 10 online groups and individuals. We then used thematic analysis and drew on principles of co-production (equality, diversity, accessibility, and reciprocity) as a deductive coding framework to analyze our findings.

**Results:**

Our findings point to distinct patterns of feature use by different people involved in care of elderly people. This diversity makes possible the principles of co-production by offering equality among users, enabling diversity of use, making experiences accessible, and encouraging reciprocity in the sharing of knowledge and mutual support. We also identified that explication of common resources may lead to new forms of competition and conflicts. These conflicts require better management to enhance the coordination of the common pool of resources.

**Conclusions:**

SM uses afford new forms of organizing and collective engagement between patients, carers, and professionals, which leads to change in health and care communication and coordination.

## Introduction

### Background

Health and care in the European Union faces the triple challenge of aging, fiscal restriction, and inclusion [1]. In the United Kingdom, the number of elderly people will increase to 6.6 million over the next 25 years. In Scotland, by 2035, the 65+ years age group is projected to have grown by 82%. This study focuses on three important problems related to the aging population in Scotland and the wider United Kingdom. The problems are (1) an increase in population of elderly people; (2) insufficient resources to meet the health and care needs of the elderly population; and (3) social exclusion of the elderly. These lead to an increased need for government expenditure to provide and deliver health and care services, as well as an increased need for expenditure by elderly people while their income is static or falling.

The statistics show the needs of elderly people are growing, and there is an increased requirement for carers [1]. Currently, the population of informal carers is more than 10% of the 65 million population of the United Kingdom. It is projected that this number will increase to 9 million of 73.2 million (around 12% of population) in the next 25 years. The current value of care is worth an estimated £132 billion per year—approximately equal to the total annual cost of health spending in the United Kingdom, which was £134.1 billion in year 2014-2015 [2]. So an important challenge is how to resource care and health of elderly people in the future. Depending solely on economic growth to fulfill the finance needs of public services is unlikely to meet the need in a time of austerity and will inevitably lead to poorer quality of service and outcomes. Hence new ways of meeting the need for health and care are needed [3]. To reshape service delivery, we need to consider how to utilize diverse resources.

The health and care system in the United Kingdom and Scotland is being reformed. The Scottish government has announced the need for better coordination and integration in this process [4]. Examining the concept of co-production is an initial step in reforming the service delivery. Boyle and Harris [3] from the New Economics Foundation give a definition for co-production:

Co-production means delivering public services in an equal and reciprocal relationship between professionals, people using services, their families and their neighbors. Where activities are co-produced in this way, both services and neighborhoods become far more effective agents of change.

There are a range of perspectives on the production and use of health and care services. A critical aspect of such services is the governance of their production and use. In this context, one strong standpoint sees health and care resources as “common pool resources” [5]. Common pool resources [5] refers to:

A system that is sufficiently large as to make it costly (but not impossible) to exclude potential beneficiaries from obtaining benefits from its use. To understand the processes of organizing and governing CPR [common pool resources], it is essential to distinguish between the resource system and the flow of resource units produced by the system, while still recognizing the dependence of the one on the other.

This common pool of resources may involve patients, informal carers, social carers, volunteers, professional carers (caregivers), and health professionals who can be seen as co-producers of health and care services. In this paper, we focus on informal carers, volunteers, and patients and examine how this large pool of informal carers and patients could, with more careful utilization, further augment the effort devoted to care in the United Kingdom. Current public services are poorly equipped to exploit the potential social economy of family and neighbors.

The full participation of informal carers in the co-production of health and care has the potential to play a significant role in the sustainability of health and care delivery. A pressing issue is how to coordinate this massive resource with the formal health and care system to enable true co-production of health and care. This massive resource is spatially dislocated and temporally uncoordinated and engaged in responding to very local demands. Modern information and communications technology (ICT) is viewed as a key enabler to overcoming such obstacles.

Increasingly eHealth and care services is viewed as the tool to reshape health care systems [6]. We propose that, in particular, social media (SM) can be viewed as an enabler for co-production. Communication is a key element in co-production that enables coordinating across various boundaries. SM cuts across boundaries, its use is well understood, but its effects are much more poorly understood. Therefore, this paper focuses on how SM enables this coordination.

To explore the role of SM in the context of co-production (with carers, patients, and volunteers in focus), we use Cahn’s framework as our analytical lens. Cahn [7] identifies the following principles as the elements that put co-production into action:

- Equality: no group or individual is more important than others. Everyone is equal and they have assets to contribute to the whole.
- Diversity: diversity and inclusion are important principles in co-production. So, diverse groups must be included.
- Accessibility: if everyone is going to take part on an equal basis, then everyone needs to have the same opportunity to be involved in activities, in a way that is suitable for them.
- Reciprocity: When people put in effort to contribute, they need to feel valued as well as needing to receive something back. This means that everyone is responsible and they have expectations, and therefore it is a mutual process.

Although these are critical elements of co-production, achieving all of them at the same time may result in asymmetry (among the elements) or conflicting goals. For instance, in some cases, encouraging inclusivity and diversity (having a large number and more diverse actors involved in one space) may be at the cost of equality and reciprocity (not everyone contributes equally or at all times). Therefore, in this paper, we initially highlight how SM enables these four elements, and then we discuss the possible conflicts.

By using this framework, we foreground the communication aspects of SM. We recognize this as one of the numerous aspects of co-production. In particular, further research is needed to explicitly heed to issues of resourcing, conflict and competition for resources, and the overall governance of health and care provision. Our focus here, therefore, is on the communication and cooperative utilization of health and care resources among patient representatives, carers, and volunteers. We will therefore discuss its limitations in the Discussion section and address the broader aspects and possible contentions involving health professionals and social workers in a later paper. SM are online tools for the creation and sharing of digital content. They aim for widespread use and are capable of supporting an unlimited number of users.

**Figure 1:**
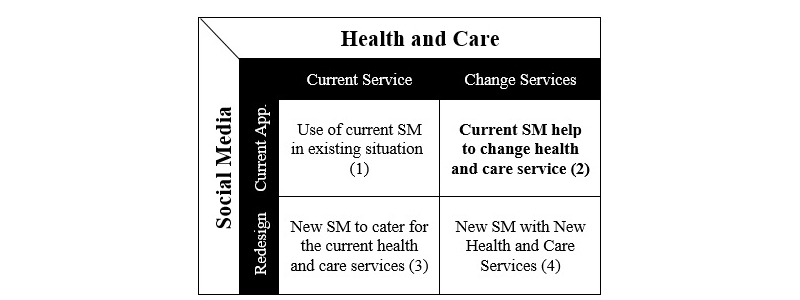
Social media (SM) and health and care.

Kaplan and Haenlein [8] defined SM as “a group of Internet-based applications that build on the ideological and technological foundations of Web 2.0, and that allow the creation and exchange of user generated content.” Dissemination of content operates at Internet speeds. It has been argued that SM has caused a change in social action in many areas [9,10]. SM increases social interaction between patients and health organizations. Moorhead et al [11] explain that SM is a powerful tool for collaboration between users, and it acts as a social interaction mechanism for its wide range of professional and nonprofessional users. It empowers public and patients by enabling them to communicate with each other and exchange health information [9,10,12]. It enables users to discuss sensitive issues [13,14]. Moorhead et al conducted a systematic review of uses of SM for health and show that SM offers peer, social, and emotional support for its users. They also demonstrate that SM increases interactions for patients, their families, and their friends.

The term was coined by Shipley after his research and reports on technology trends [15]. SM has since become media for the creation and maintenance of connection and interaction among individuals [16,17]. They are currently used widely by a diverse range of users and have become among the largest number of most visited sites worldwide [18].

Several studies [8,19,20] categorize SM into 9 groups: (1) Wikis; (2) blogging; (3) microblogging; (4) content communities; (5) forum; (6) instant messenger; (7) social network sites; (8) mobile SM; and (9) virtual world and online social gaming.

In health and care, we divided SM to 3 groups: (1) SM that were created for general-purpose use and is now used for health and care, for example, Facebook groups (FBGs); (2) SM that were created for health purposes and make use of generic SM for other purposes (eg, 3D-Doctor or some other health applications (apps) that make use of Skype to connect patients to the doctors); and finally (3) apps created for health and care purposes that use the concept of SM (Health SM), for example, HealthUnlocked is new SM for health purposes [21].

### Aim

The aim of our study was to investigate the current and possible future for SM as an enabler of co-production in health and care for elderly people. To achieve this aim, 2 main sets of questions are asked: (1) What are the uses of current SM in enabling (and reshaping) health and social care? and (2) How can SM be reshaped to enable (and reshape) health and care co-production?

We consider a typology of opportunities and limitations of SM for health and care. Figure 1 shows existing health and care service bundles with (1) existing or (2) new SM tools (new SM means some app developed for health and care that used the concept of SM such as HealthUnlocked). New health and care service bundles with (3) existing or (4) emerging SM tools. In this part of the research, we focused on (2): “How current SM help to reshape or change health and care services?” In other words, we assessed how existing SM acts as an intervention during the reshaping of health and social care in the United Kingdom by enabling co-production (in particular, co-services).

## Methods

### Overall Project

This paper focuses on one of the four aspects of a larger qualitative study that investigates the sociotechnical aspects of the current and possible future uses of SM by different organizations and groups of health and social care as an enabler of co-production in the United Kingdom, in particular Scotland. We conducted 20 semistructured interviews, which focused on the services offered, the types of online apps (particularly SM) used, their challenges, and the future possibilities of SM. We used purposeful sampling to select organizations and groups that were providing care services to elderly people. We used a combination of interviews and analysis of the activities and content of online groups to collect data. Combining different methods enabled us to triangulate the data sources to validate our findings.

### Material and Methods

This paper focuses on the second section: How current SM help to reshape or change health and care services? (Figure 1). Our appraisal adopts a sociotechnical technique [22,23], using a mixed-methods framework, including multiple methods (interviews, observations of online activities, and secondary data analysis) and multiple sources of data. Table 1 summarizes the data collection methods and sources. For the purposes of anonymity, names have been replaced with pseudonyms.

### Theoretical Framework

Normalization process theory (NPT) has been used as our theoretical framework to enables us to obtain meaningful understanding of the complex sociotechnical processes involved in the use of SM tools and service within health and care co-production. NPT offers a whole system perspective, to assist researchers to make sense of the social and organizational aspects of different interventions and to better conceptualize the complex adaptive systems. NPT, which has been used in many eHealth research studies, has been used as a tool in this research to assess the changes brought about by the introduction of SM into the personal and organizational lives of patients, carers, and organizations involved in care activities (Table 2).

### Data Collection and Qualitative Data Analysis Procedure

We conducted 20 interviews (approximately 22 hours) with patients, carers, and employees of third-sector or intermediary organizations (ie, charities), which provide funds and services for developing programs to reshape of health and care services in Scotland, and companies or organizations working in the health sector providing services to elderly patients with long-term conditions.

We used purposive sampling to select the interviewees. Our purposeful sampling strategy aimed to identify organizations that actively used some type of SM in their activities. The selection criteria for organizational participants was people who were either involved in providing carer activities, decision makers, or those involved in design of ICT programs for elderly care. For nonorganizational participants, we aimed to select interviewees who were either patients or carers who actively used some type of SM in their day-to-day life.

We used the NPT framework to develop an open-ended interview question guide (Table 3). The interview questions were tailored to the roles of individuals and further refined throughout the research based on the findings of prior interviews. To complement this data, we used secondary data, generated by one of the abovementioned organizations, about uses of SM in self-management. The data consisted of eight interviews with people with long-term conditions who used SM for health purposes. Data from these interviews were analyzed together with the primary interview data.

**Table 1:** Summary of primary data collection (X indicates inclusion).

Number	Name	Description	Interview	Observation of online activities			
				Twitter	Facebook	Website	Others
1	Organization 1	Professional sector	X	X	X	X (Web 1.0)	
2	Organization 2	Intermediary	X	X	X	X (Web 1.5)	Blogs
3	Organization 3	Intermediary	X	X	X	X (Web 1.0)	LinkedIn, Instagram, YouTube
4	Organization 4	Intermediary, part of a larger project	X	X	X	X (Web 1.0)	YouTube
5	Organization 5	Intermediary, part of a larger project	X	X		X (Web 1.5, 2.0)	Blog
6	Alison Morgan	Project manager (FBG^a^ admin)	X				
7	Sarah	Patient (forum and FBG user)	X				
8	Edmund	Patient (forum, FBG, YouTube, and video blog user)	X				
9	Carole	Patient (forum, FBG, and charity website user)	X				
10	Donna	Patient and carer (Forum, FBG, and voluntary organization website user)	X				
11	Karen	Carer (forum and FBG user)	X				
12	Laura	Patient and carer (Forum and FBG user)	X				

^a^FBG: Facebook group.

**Table 2:** Representation of the 4 constituent normalization process theory (NPT) constructs that attend to the 4 key aspects in eHealth implementation.

NPT constructs	Coherence (sense-making work)	Cognitive participation (engagement or buy in work)	Collective action (enacting work)	Reflexive monitoring (appraisal work)
Questions	What gets done with social media (SM) in co-production? What gets done with other mechanisms?	How does SM facilitate participation within the intervention?	If an actor did not have SM, what would happen to his or her work (in terms of quality of service delivery)?	Does SM allow participants to reflect on the work they have done?

**Table 3:** Normalization process theory coding framework used for qualitative data analysis.

Coherence (sense-making work)	Cognitive participation (engagement or buy in work)	Collective action (enacting work)	Reflexive monitoring (appraisal work)
Differentiation: What gets done in social media (SM)? What get done in other ways? What are the overlaps?	Enrollment: Can actors articulate the benefits of SM?	Skillset workability and Interactional workability: What do the users communicate through SM? To what extent does SM support co-productive work?	Reconfiguration: Do third party or charity organizations reflect on their activities on SM to develop new services through use of SM with co-production? Does reflection on SM contribute to redesign?
Communal Specification: How does SM contribute to the work? Do people agree with this as an account of the collaboration?	Activation: Can actors articulate how their work will change? Are they positive about this?	Contextual Integration and Relational Integration: When users contribute in SM argument, does this have any influence on the decisions made? How does SM activity get captured and reused?	Communal Appraisal: How does SM influence coordination between organization and individuals in this context? Does SM let people build groups which are effective in service delivery?
Individual Specification: What does each actor use SM for? How is that different from what other actors do?	Initiation: Do actors understand their new activities involving SM and are they happy to conduct them?	Interactional Workability and Skillset Workability: How do responsibilities change?	Individual Appraisal: How do individual carers or service users appraise the effects of use of SM on them and their environment?
Meaning (internalization): What would be lost if SM were not used?	Legitimation: To what extent do actors and organizations believe that the action involving SM are important to the provision of the service?	Relational Integration and Contextual Integration: How does SM change the resource flow?	Systematization: How do organization (third sector or voluntarily) or individual users of SM in this context determine the effective (benefits or risks) or usefulness of SM in this context.

Finally, we observed the online activities of interview participants (organizational participants) and their uses of SM for health purposes. This enabled us to find evidence and complementary data to support the claims. Table 1 provides a complete list of the observation sources for each of the participants.

Data were collected over the period from March 2015 to December 2015. All conducted interviews were transcribed verbatim and transcripts checked for accuracy. We continued data collection until we judged that no new themes were identified and saturation was reached [24].

### Data Analysis

Data were coded in NVivo software version 11 (QSR International) and thematically analyzed for each type of SM. We drew on the four principles of co-production (equality, diversity, accessibility, and reciprocity) as a deductive coding framework, extracting excerpts from our qualitative data that had bearing upon how SM reshapes co-production. In addition, we also inductively identified emerging themes surrounding the benefits and challenges of SM in enabling co-production in health and care, which served as an analytical lens to examine our data using a deductive approach to analysis [25]. Negative cases, that is, those that did not fit within the narrative, were explored in the most detail.

### Research Governance and Ethics

This study was granted ethical approval by University of Edinburgh, School of Informatics. Consent forms were signed and agreed by all participating respondents. Identities were protected and assigned a confidential generic descriptor to ensure anonymity, and all names were changed.

## Results

Our findings show that overall existing SM helps support the four principles that underpin co-production—equality, diversity, accessibility, and reciprocity—and will influence the informal care sector to become more efficient. Below we explain how each principle of co-production can be enabled by existing SM. While appreciating the benefits, we also found tensions caused by use of SM as well as challenges that inhibit use of SM for co-production.

### Equality Through Sharing Experience of Users as Valued Assets

To enable equality, individuals need to have the same status within a group and the group needs to recognize the value of the contribution of all individuals. Some types of SM (in particular, private FBGs) seem to allow recognition of skills and abilities of all members within a group.

Private FBGs were widely used by people who wanted to be connected to each other in a secure and closed manner. Participation in these groups needed to be approved by the administrator(s) based on whether individuals are patients or carers of a person with a particular condition. Therefore, those who were members of these groups held experiences, skills, or abilities in dealing with the condition. This knowledge was recognized by others as an asset that could be shared leading to a sense of being valued by others:

My knowledge is useful for others and their experience is valuable for me. We talk about our condition and liaising with each other and find ways to deal with issues...one particular case was when I had an issue in using my glucose meter and I found I was doing it wrong. I could’ve waited to see my GP, but got the answer in the group.

Patient and Carer

These experiences and skills either facilitated knowledge exchange or provided mental support, which in either case were seen as important to the group members. There are clear considerations of empowerment when people feel that their knowledge and skills are contributing to a change in the world. Although many positive consequences exist, we also need to be aware of the issues that may arise from this knowledge sharing and empowerment. These issues include the extent to which knowledge leads on to changes in the productivity of the health and care system (and possible lack of applicability of knowledge for some members of the group) and the means to prevent inaccurate or harmful information from propagating through the network. In similar terms, health and care professionals express concern over the unregulated transfer of experience through SM, which leads to a need for filtering and integration of information in such groups.

In many cases, the administrators of the groups also had the same condition as other members (or were the carers of people with the same condition). Having the condition meant that they were also equally concerned about the surrounding issues and had dealt with them for a considerable time. Thus, on the one hand, they brought comparable assets to the group, and on the other hand, they were equal in terms of status and position:

...with a closed group, you could have a moderator or an admin who works with that condition, so...they are going to actually facilitate the whole group, and without their, service provision, that group wouldn’t exist, and often the closed groups are not run by charities, they might just have been set up originally by someone who has had that particular experience, and they feel that there is a community for them of people in their situation out there, so they set it up themselves.

Patient

As a result, although the member of these groups appreciated the equality of status, a new tension was created. Patients and carers acquired a considerable knowledge that could stand alongside health professionals; however, by no means were they equal in status or position to them. This in turn could lead to conflicts between the 2 groups.

This equality in terms of condition and experience removed the culture of “them and us” [26]. This, in turn, led to higher levels of support between all members (including administrators):

...they are volunteers who live with the condition, not employees of any organization.

Patient

This was achieved by the closedness of the group (to ensure participants have similar levels of experience). However, this closedness could lead to tensions in terms of accessibility and diversity elements (discussed in the next section).

This equality in FBGs has empowered users to talk openly about their professional care practices and even discuss and find ways to approach professional carers (eg, general practitioners, National Health Service (NHS) consultants):

I definitely feel more in control too. For example, I was fobbed off a couple of years ago when asking a doctor for Vagifem and he said to use KY Jelly. The ladies here gave me the confidence to go back to my usual GP and ask assertively for the Vagifem I knew I needed. He agreed that Vagifem was a good idea and has prescribed it for me ever since.

Patient

So, in general FBGs (and forums) generated a sense of community that facilitated equality among its users. However, there were times that things did not go as smoothly. Some members were aggressive about the stance they took on issues, which could lead to disagreement or, in more extreme cases, abandoning of the group:

Some people are militant when talking about their stance pro-anti surgery for Colitis and Crohns. They’ll really push their ideas on people and be very hard to talk to. You might have one person claiming to have the perfect solution to your problems: “Just cut out dairy!” Or someone else claiming that surgery or medication is a con by the health professionals. With Colitis and Crohns there are such extremes of symptoms and illness and a lot of people are frequently misdiagnosed due to this.

Patient

### SM Enables Diversity by Being Inclusive of Underrepresented Groups and by Connecting Diverse Groups of People

Diversity was enabled by SM in two ways. First, patients and carers are diverse in terms of characteristics (eg, literacy) and conditions. These differences can lead to less ability to access and use resources. Inclusiveness means overcoming these diversities and making sure that the people who are less likely to access or use resources are by some means gaining the benefit of these resources.

Patients mentioned that the closed nature of some SM, in particular, the private FBGs and forums, gave them the ability to talk about issues that cannot be discussed face-to-face because of embarrassment about conditions of particular illnesses. This meant that some of those who were formerly excluded because of their conditions could now benefit from these discussions:

People are more open about their experiences because it’s a closed group. They feel more open than if it was in the public domain...Online support takes away a lot of the social difficulties of sharing in a group for fear of embarrassment or sounding stupid.

Patient

On a forum you talk about how you really feel, without any of the normal taboos. You can talk about anything.

Patient and Carer

However, although this closeness of forums was an effective factor in facilitating some of these talks, it also created the challenge of getting into the groups. Thus, this closedness was a drawback as individuals could not join the groups without the permission of the administrators.

SM was not able to overcome many of the other barriers. For instance, interviewees highlighted that not everyone could have access to various SM types such as FBGs and forums. This could be due to limited Internet access or low technological literacy.

Second, some types of SM, such as Twitter, acted as an effective place for connection of diverse people in health and care sector, including professionals and nonprofessionals (carer and patients). In comparison with many other SM, Twitter was used by a larger number of professional people:

I think generally Twitter has certainly helped us to increase the amount of people that we have on the network. And also, to increase the amount of people that come along to the events. But again, we feel that that’s mostly in that professionals. So, we don’t really think that it’s been helpful in terms of targeting people with long-term conditions or carers at the moment.

Organization participant

As Twitter is a rapid and flat SM app, it provided a good space for users to find answers to their questions (without necessarily having to connect directly with people), getting current information and keeping up-to-date with health news:

I think Twitter been used for exchange of informal information and really really useful information around about research. I found it extremely useful for the work on health literacies...So, you get to know people who are working and developing interesting stuff from health literacy...Twitter is good for following and that keep yourself up to date.

Organization participant

The flat nature of Twitter (no connections needed) also provided a good platform for raising funds or promoting campaigns by organizations and charities. In doing so, organizations used Twitter to promote their activities and keep all users updated. This is illustrated in the quotes below:

...so, we’d be very keen to promote our work [on Twitter], so we make sure that they're linked to, we would be promoting. [Organization participant].

...it’s useful for campaigns as well, so there’s been a lot of really effective health campaigns on Twitter.

Organization participant

However, issues, such as filtering imposed by the NHS in the use of SM on its premises, led to limitations in the use of such apps. One participant explained that their organization set up a blog; however, its use was constrained because of the firewall introduced by NHS that blocked access to blogs during daytime for professionals:

...there are a massive [number of] health care staff using social media throughout our day but firewall is a big problem.

Organization participant

So, although SM enabled diversity in terms of opening up a space for communication and knowledge sharing of some patients (and carers) with particular conditions, as well as offering a fast and flat platform for various actors (including health professionals, social workers, and carers) to share news, there were yet many barriers that limited the use of SM. As highlighted by the participants, individuals who had Internet accessibility issues could be excluded from gaining the benefit from SM. This could be either because of limited Internet access or the inability of some elderly people in using technology.

### SM Makes Groups’ Experience Accessible

To allow accessibility means everyone should have the same opportunity to participate in activities in a suitable manner. By offering various types of platforms (eg, blogs, FBGs, and Twitter), SM allowed different individuals to take part in knowledge sharing and communication in a way that suited them best:

The one thing we found about Twitter, it seems to be very much used by the professionals. We find that most people with long-term conditions and carers will use Facebook. Whereas with Twitter, we will seem to target lots of professionals.

Organization participant

This allowed patients to gain access to some of the resources that were shared by professionals. Although it helps them reach a new layer of information about particular conditions, this did not mean having direct access to knowledge that leveraged their own condition. Therefore, accessibility was enhanced to some extent and for some of the users only.

Moreover, accessibility to group experience is enabled for those who have difficulty to gain access to others’ knowledge otherwise (such as through face-to-face meetings):

I have quite a bad chest as you can hear, so I can be spending a lot of time on the forums or groups when I’m shut up in the house.

Patient

This accessibility to knowledge from various sources, in turn, empowered users, as illustrated by the quotes below:

I would say that social media certainly empowers you. By people sharing their experiences, it makes you far more informed. You can find out what kind of treatments are out there and go to appointments armed with information. I also felt more empowered in how I dealt with health professionals if I felt I wasn’t being listened to. In fact, I later lodged a formal complaint to the health board.

Patient

I’ve just had my results in from my test. GP, I saw him 2 times, never once told me that these results—and they were bad results. The GP missed it.

Organization participant

Although it increases patients’ knowledge, this was not necessarily welcomed by all professionals. Some professionals preferred to guide patients’ knowledge in certain directions. They believed that this knowledge is partial, and it will either lead to loss of trust or “interfere” with the course of their treatment (if patients take the advice from other sources rather than their direct healthcare professionals). They also believed that this knowledge does not take account of other issues such as limitations in NHS funding. Therefore, it can lead to new conflicts in terms of accessing scarce resources.

Another difficulty mentioned by patients was excessive online accessibility. This referred to the fact that sometimes too much online activity could lead to reduced physical activity. In more extreme cases, patients stated that too much focus on the negative comments of others could lead to discontentedness:

Plus, you’ve got to watch that you don’t get too immersed. You could easily spend all your time on Facebook, or on Forums.

Patient

And just talking to people about their illnesses might get you down.

Patient

To reduce some of the negative effects of SM use, some organizations (such as charities) introduced content and structure “configurations.” So, at the same time as giving a space to patients and carers to be active in sharing their stories, they would also put a control on what was shared and how it was shared.

We’re generally asking people about their story. And to share our story through our blog. So, we have like a set guideline for it. We will send people a guideline on how to write a blog, give them the word limit of the blog, and what kind of content it’s good to have in a blog.

Organization participant

However, such controls were costly to manage as organization members had to spend time going through each post and modifying them to meet the organizations preset framework. To manage this, some organizations used means of co-production by putting people with experience of effective post writings in touch with the newcomers to help them produce content, which was fit for the purpose.

### Reciprocity SM Encourages Reciprocity in Sharing of Knowledge and Mental Support

Reciprocity refers to the mutual process of giving and receiving something back. Users of SM, in particular FBGs and forums, emphasized that they expected to gain something back from the group. Reciprocity may be direct (members behave in response to other members’ acts) or indirect (cooperation with strangers to gain reputation) [27]. Direct reciprocity could be generally seen in offering knowledge and experience about a topic:

Using social media is actually pretty empowering. When I was diagnosed, I had to become an expert on the condition and there’s no better source of knowledge for this condition than your own lived experience. I did a lot of personal research: first asking doctors and nurses about it, but the best information comes from the women who live with it.

Patient and Group administrator

Indirect reciprocity, on the other hand, could be seen in offering mental support:

I wouldn’t want to join a group unless I thought that people would be able to empathize and understand what I’m going through. There’s no point in talking to people who don’t understand- they won’t respond appropriately.

Patient

The sympathy that came from patients with similar health conditions (rather than paid organizational members) created added value for its recipients and led to the creation of a positive relationship:

The knowledge and information comes from the members of the group. It’s the people living with the condition who have the experience of self-managing, not paid employees of a charity who don’t necessarily live with a condition.

Patient

Both forms of reciprocity played an important role in keeping the communities going. Therefore, administrators encouraged members to participate in talks, to make sure that everyone is receiving something back from the group.

We ask people to be active participants in the group: to commiserate with each other on a bad day, to be supportive of each other and share knowledge and experiences.

Patient and Group administrator

Some administrators went further by deleting the members who were not active for a certain period of time:

People who don’t participate for a more than a couple of months are deleted from the group.

Patient and Group administrator

However, lack of involvement in discussions was sometimes due to lack of knowledge in the topic area or disagreement with the stance taken by other individuals. Therefore, administration of groups was a challenge:

Even if I don’t comment on posts, I read them so that I may be aware of any issues I may face...I don’t like the idea of taking HRT (Hormone Replacement Therapy) or any other things like creams and stuff. I prefer the natural route but I do understand now with information posted that each individual has their own opinions on the matter. These opinions and choices are personal to them and I take that on board now because this information is important knowledge.

Patient

So although reciprocity was important in terms of the overall activities of individuals, the administrators needed to be considerate of members with lesser contributions. In some cases, some patients and carers started their participation as lurkers, just to get a feeling about the environment or to gain some specific knowledge. It would then take some time for them to reciprocate to the group. Therefore, user engagement could be seen as a gradual phased process. For those people with lower levels of engagement, who would be passive readers, it could begin by encouraging them to read more regularly, then starting to comment, and then contributing. The use of SM creates the opportunity to allow for growth of continuous knowledge and emotional conversation of strangers *.*

## Discussion

### Summary of Findings

This work indicates how different types of SM enable co-production by supporting its underlying principles: equality, diversity, reciprocity, and accessibility. The paper also offers insights into the challenges involved in use of these SM as an enabler of co-production. Individual users (patients and carers) and organizations providing health care services to elderly people adopted various kinds of SM to meet their diverse needs. We observed that people’s contributions evolved as they became more experienced in the use of SM. Table 4 summarizes the benefits of each type of SM in terms of co-production principles.

In general, private FBGs were the most widely used SM by patients with similar conditions and their carers because of their greatest offerings around: (1) equality of members and valuing their experiences as assets; (2) diversity and inclusion of members whose voices are less heard otherwise; (3) accessibility for people from different geographical locations; and (4) reciprocity of knowledge sharing and mutual support. Forums were similar in terms of benefits and use; however, they were mainly sponsored (and administered) by organizations. This allowed for better control of data; however, their formation and access were more challenging. Microblogging (eg, Twitter) was also seen as one of the most highly used SM apps, which plays a very important role in health and care by both professionals (eg, doctors) and nonprofessionals (patients). Its “flat” nature allowed rapid exchange of information based on users’ interest in topics. Therefore, patients needing information or updates about particular diseases could easily gain access to information shared by health and care professionals. It was also highly used by those who wished to attract communities of interest or funds or those who wanted to provide or receive fast update about news and various topics. Therefore, it served for a very different purpose to those of FBGs and forums. Blogging, on the other hand, was used for slow but detailed sharing of stories by people and organizations about their health interests and experiences. We found four affordances of SM that supported care for elderly people: knowledge creation and sharing, information dissemination, emotional support, and new communication channels. SM afford behaviors that were difficult (or impossible) to achieve before these new tools were used by those involved in the care of elderly people. We further found mechanisms that affect how people engage in the knowledge and support conversation, which may have positive effects or may result in adverse consequences not intended by the participants or other groups involved in care of elderly people. These emergent tensions are the basis for the implications we draw.

In this way, SM offered new modes of communications not only between patients and their carers, but also between them and the professionals. On the one hand, professionals gained access to patient stories (blogs, FBGs, and Forums) and the details of conditions. This information can be used by doctors for better diagnosis and monitoring of particular patients. On the other hand, patients and carers gained access to new health and care findings.

Also, the joint effort in the creation of and monitoring of knowledge contents as well as the self-promoting nature of SM improved the productivity of health and care organizations by enabling them to publicize information using low-cost mediums.

### Interpreting Findings in the Context of the Wider Literature

The large body of extant studies around the use of SM for health and care focus on who uses these tools [17,28-31] and uses of SM for communication [11,32-34]. The studies show that SM increases patients’ and carers’ access to health information [14,35-44]. Although our study confirms this, we specifically show that SM makes various types of health and care resources visible to meet the needs of elderly patients. These resources include availability of carers (including professional and nonprofessional resources), care programs (eg, outgoings, charity programs), knowledge about symptoms and cures of different conditions (including diets and drugs), new communication techniques with professionals, and more. We show that by facilitating new modes of dialog between different actors (ie, patient-patient, patient-carer, carer-carer, patient-professionals, and patient-healthcare organization), SM enables new, faster, and more effective modes of social interactions in which patients become empowered by having access to more resources.

SM offers a wide range of benefits for health communication, which can be grouped into increased interaction around general [17,45] and sensitive information [13], better accessibility of information [17,32,33,44,46-52], and emotional support [10,13,40,53-61]. We use Cohn’s co-production framework to expand the extant findings by showing how such characteristics act as the key principles of co-production. Our work shows that SM enables recognition of the experiences and skill of all participants as assets and enables them to engage with the community and become active.

**Table 4:** Social media for co-production.

Co-production principle	Equality	Diversity	Accessibility	Reciprocity
Facebook groups	Patients with same condition and their carers; Experience and skills seen as asset	Less heard voices are included	Members from diverse geographical locations	Mutual support; knowledge sharing; administration of participation
Forums	Patients with same condition and their carers; Experience and skills seen as asset	Less heard voices are included	Members from diverse geographical locations	Mutual support; knowledge sharing; administration of participation
Microblogging		Professionals and nonprofessionals; No direct connections needed	Retweets; provides access to another social media	
Blogging			Accessible by all	Feedback on blogs

Our study also expands the existing literature, by showing that the fulfilling of different needs by various SM is influenced by different factors, including the speed of knowledge creation and dissemination, the speed of feedback and discussions, the detailed nature of knowledge exchanges, the type of discussion (support vs news vs health knowledge sharing), and the openness and closedness of activities. These characteristics help better coordination and communication of knowledge resources between carers and patients.

There are also limitations in the use of SM. Information quality concerns and the lack of reliability of the health information [10,38,40,41,45,53,62-69] are among the widely discussed limitations. Although our findings confirm these, we also show that the explication of common resources may lead to new forms of competition and conflicts. In particular, the new knowledge that is obtained by users is not always welcomed by professionals. This could be due to numerous reasons, including lack of validity of all information obtained, as well as higher demand for treatments as they become known to patients and carers. Also, because of concerns about information quality and validity, some health care organizations need to put into place new forms of information monitoring, which may be costly.

### Strengths and Limitations

This paper has a number of strengths and limitations. We drew on NPT [23,70], which served as a sociotechnical analytical lens, to help us analyze the benefits as well as challenges of various types of SM. We have drawn data from multiple different sources, including patients, carers, and charity organizations to enhance confidence in our findings and included diverse perspectives. However, because of the sensitivity of patient data, we only had limited access to private FBGs and forums. We overcame this problem by contacting many groups and gaining access to one particular group. To also understand other groups that were important for this research, instead of observations, we interviewed its users. We also did not seek the perspective of NHS professionals including doctors. This can be addressed in future research with a focus on professionals. Finally, in this paper, we have focused on communication and cooperative utilization of health and care resources. Therefore, further research is needed to focus on resourcing, conflict and competition for resource, and the overall governance of health and care provision.

### Conclusions

SM has gained momentum within the health and care community by offering significant benefits for patients, carers and even professionals; increasing interaction; providing more readily available and customized information; offering mental support; promoting health and care–related activities; offering a platform for communication for underrepresented individuals; allowing reciprocal sharing; and enhancing the communication between patients, carers, and professionals. All these benefits have the potential to be realized through SM. These benefits facilitate co-production by enhancing equality, diversity, accessibility, and reciprocity, and lead to recognition of resources (skills and time), joint creation and monitoring of knowledge, and direct and indirect mutual support. This in turn can lead to resource savings needed to manage the growth in demand from the expanding elderly population. SM allows users to learn from each other (in a less costly manner) and can facilitate communication more effectively (in particular, professionals and nonprofessionals).

However, despite these benefits in facilitating co-production, existing SM does not fully enable co-production. There are as yet outstanding issues in arranging the common pool of health and care resources to better enable co-production. Different SM enable co-production (co-delivery) of services for elderly people to varying extents. In particular, SM is used distinctly differently by professionals and nonprofessionals. This can be seen as an opportunity to leverage their benefits in a more productive manner.

## Abbreviations

FBG: Facebook group
ICT: information and communications technology
NPT: normalization process theory
SM: social media
NHS: National Health Service
